# Valuation of Life Among Old and Very Old Adults: Comparison Between Germany and Japan

**DOI:** 10.1093/geroni/igy020

**Published:** 2018-07-11

**Authors:** Takeshi Nakagawa, Daniela S Jopp, Yasuyuki Gondo, Jonathan Lehrfeld, Christoph Rott, Frank Oswald

**Affiliations:** 1 Section of NILS-LSA, National Center for Geriatrics and Gerontology, Aichi, Japan; 2 Japan Society for the Promotion of Science, Tokyo, Japan; 3 Institute of Psychology, University of Lausanne, Switzerland; 4 LIVES, National Centre of Competence in Research, University of Lausanne, Switzerland; 5 Graduate School of Human Sciences, Osaka University, Japan; 6 Department of Psychology, Fordham University, Bronx, New York; 7 Institute of Gerontology, Heidelberg University, Germany; 8 Interdisciplinary Ageing Research, Goethe University, Frankfurt, Germany

**Keywords:** Culture, Old-old, Quality of life, Young-old, Well-being

## Abstract

**Background and Objectives:**

Valuation of life (VOL) represents a construct capturing individuals’ active attachment to their life. The majority of studies on VOL were conducted in North America and Europe where personal autonomy and independence are highly valued, leaving open the question about the relevance of this construct in interdependence-oriented cultures. Using a framework of cross-cultural and life-span theories, the present study compared levels and predictors of VOL between the young-old and old-old individuals from Germany and Japan.

**Research Design and Methods:**

Two hundred fifty-seven Germans and 248 Japanese, matched by age, gender, education, and IADL, answered a 5-item VOL scale and shared information on sociodemographic, social, and health resources.

**Results:**

Germans’ VOL levels were higher than in Japanese participants. Both culture- and age-moderated predictions of VOL: education was significant only in the young-old Japanese, and close social partners mattered in the old-old, not in the young-old. Health determined VOL irrespective of culture and age.

**Discussion and Implications:**

The findings suggest that cultural values and aging processes should be considered to better understand how individuals value their life and to help older adults to feel that his/her life is meaningful and worth living.

**Translational Significance:** This study highlights the role of cultural values and aging processes in how individuals perceive their life as meaningful and worth living. We show that social status matters more among older individuals in an Eastern culture compared with those in a Western culture, and that close social partners are more important in very late life. Professional care providers and family members will benefit from these findings to improve older adults’ positive appraisals of their life.

## Background and Objectives

Given worldwide increase in life expectancy and exponential growth of the very old population, investigation of individuals’ perceptions of the value of their life when faced with age-associated multimorbidity and frailty is of high actuality. Lawton and colleagues (1999, 2001) proposed the concept of valuation of life (VOL), to capture older adults’ positive appraisals of their life. VOL was defined as an active attachment to life, and the total score indicates the degree to which the individual feels his/her life is meaningful and worth living. Empirically, Lawton and colleagues (2001) revealed that VOL was related to positive psychological functioning such as well-being (e.g., psychological well-being and self-esteem) and personality (e.g., optimism and mastery). In addition, VOL can be useful in planning end-of-life treatment because the construct was associated with behavioral outcomes, such as how long people wished to live under hypothetical health scenarios (Lawton et al., 1999). More recently, VOL was recognized as an important outcome in gerontological research with potential implications for advancing psychosocial interventions (Gitlin, Parisi, Huang, Winter, & Roth, 2016, 2018). Yet, the empirical basis regarding VOL and its associated factors is rather limited, as only a few studies have been conducted mostly in Western countries (e.g., Jopp, Rott, & Oswald, 2008; Moss, Hoffman, Mossey, & Rovine, 2007). Only Gitlin and colleagues (2016) sought to understand variations in race by comparing Caucasian and African Americans. With the exception, one study was conducted in Japan (Nakagawa et al., 2013), but cultural differences in VOL have not been examined. Given the potential role of culture in aging (Fung, 2013), however, how individuals value their life may not only depend on personal factors such as chronological age, but also on contextual factors such as cultural values. To better understand the VOL construct, the present study investigated levels and predictors of VOL by comparing old and very old individuals in two cultures (i.e., Germany and Japan).

### VOL and Culture

Culture is historically transmitted meanings (e.g., values) that are instantiated by practices (e.g., rituals), and individuals perceive or make sense of their life through their internalized cultural values (Fung, 2013). According to a cross-cultural theory of self-construals (Markus & Kitayama, 1991), independent versus interdependent values shape how individuals perceive their life as valuable. Specifically, with a more independent sense of self, individuals learn to value personal autonomy and independence from birth, and thus may consider their personal capacity of remaining healthy as important in later life. As a result, in independence-oriented cultures, independence is regarded as essential for everyday life (Mack, Salmoni, Viverais-Dressler, Porter, & Garg, 1997). In contrast, with a more interdependent sense of self, individuals learn to prioritize the needs of their social group over their own throughout life, and such cultural values buffer the negative effects of receiving support from a child on the well-being of older parents (Takagi & Saito, 2013). Consequently, in interdependence-oriented cultures, loss of autonomy may be less harmful in later life than in an independence-oriented one: being part of a social group or feeling socially connected may be of particular importance in interdependent cultures to define one’s life as valuable. Given these cultural differences in self-construals, levels and predictors of VOL could vary across cultures: whereas health resources are more important in independent cultures, social resources mattered more in interdependent cultures. Thus, age-related loss of autonomy can be more harmful in independent cultures relative to interdependent cultures, which lowers attachment to life in very late life especially in independent cultures.

### VOL and Age

While the cross-cultural theory implies that aging is under the influence of cultural values, a life-span theory proposes age-related changes which may emerge similarly across cultures. According to the socioemotional selectivity theory (Carstensen, 2006), seeking emotional closeness presents the primary social motivation when the time horizon shrinks with advancing age. Jopp and colleagues (2008) reported results in line with the theory, indicating that VOL predictors differed according to age group: multiple health indicators were predictive of VOL in young-old individuals, while only one health indicator was significantly associated in old-old individuals. In addition, for old-old individuals, more frequent indirect contacts were significantly positively associated with VOL. Therefore, empirical evidence suggests that health factors appeared to become less important, while some social factors increased in importance with advancing age. However, no study to date has examined age differences in VOL in Eastern countries, and thus the direct comparison between cultures could provide implications for generalizability of existing evidence and potential intersections between culture and aging.

### The Present Study

The main purpose of the study was to examine cultural and age-related variations in mean levels and predictors of VOL in old and very old adults from Western and Eastern countries. We compared data from Germany and Japan. Germany represents a country characterized by an individualist-independent culture, while Japan represents a country characterized by a collectivist-interdependent culture (Suh, Diener, Oishi, & Triandis, 1998). Besides, both countries are rapidly aging, and have taken similar public measures, such as comparable long-term care insurance systems, to prepare for increasing numbers of older individuals with physical and mental age-associated issues.

The present study addressed three hypotheses. First, given the cross-cultural theory of self-construals (Markus & Kitayama, 1991), we hypothesized that mean levels of VOL would be higher in Germany than in Japan, because individuals from an independence-oriented culture would strongly value their personal capacity of remaining healthy in later life. Moreover, we expected that VOL would be lower in the old-old group in Germany compared with the young-old group, given that loss of personal autonomy would be more deleterious in Germany relative to in Japan.

Second, we hypothesized that sociodemographic and social resources would be more important predictors for VOL in Japan than in Germany, as social status and relational harmony matter more in an interdependence-oriented culture. Further, we expected that the role of health resources for experiencing VOL would be more important to the Germans relative to the Japanese.

Third, we hypothesized that prediction patterns would also differ according to age group, as individuals in different life phases are confronted with different age-related challenges, which may be similar across cultures. In line with the socioemotional selectivity theory (Carstensen, 2006), social resources were expected to be more important for VOL in the old-old than in the young-old, while health resources were assumed to matter more for VOL in the young-old than in the old-old.

## Research Design and Methods

### Participants and Procedure

This study is based on data collected in two independent studies in Germany and Japan. The German sample is a subsample of a population-based study conducted by Hieber, Oswald, Rott, and Wahl (2006). In total, 773 individuals aged between 65 and 94 years were invited to participate in a mail-based survey. The study sample included 356 participants who completed the self-administered questionnaire, representing a response rate of 52%.

The Japanese sample was also based on a population-based study performed by Gondo, Nakagawa, and Masui (2013). The participants were drawn from three age cohorts aged 69–71, 79–81, and 89–91 years. A total of 2,245 individuals were invited to participate in a survey conducted in community centers. Split by age cohort, the numbers of participants were as follows: young-old age group (69–72 years): 1,000; old-old age group (78–82 years): 973; and oldest-old age group (88–92 years): 272. Response rates for the young-old, old-old, and oldest-old age groups were 23%, 18%, and 8%, respectively.

### Sample Selection

To compare the two studies, we created a sample containing 310 pairs of German and Japanese participants matched by age, gender, education, and instrumental activities of daily living (IADL). A one-to-one matching algorithm was applied to find the closest match. Given the differences in sampling frames (i.e., the age range was wide in Germany but narrow in Japan), we defined young-old, old-old, and oldest-old as individuals aged 65–74 years, 75–84 years, and 85 years and older, respectively. For subsequent regression analyses, we required a minimum of 103 individuals to detect medium effect sizes (i.e., β = .15) with α = .05 and 1 − β = .80. We thus excluded the oldest-old individuals because the sample size was too small (44 Germans and 41 Japanese, respectively) for age-group specific regression analysis. After excluding participants with missing data for the key measures, the final sample included 257 German and 248 Japanese individuals. Table 1 presents descriptive characteristics for each country. As expected given the matching procedure, there were no significant differences in gender, education, or IADL between the German and Japanese samples in chi-square tests or two-way ANOVAs with country and age group as independent factors. However, on average, Japanese participants were 7 months older relative to German participants [*F*(1, 501) = 18.22, *p* < .001, partial *η*^2^ = .04].

Whereas all the original German sample was included in the matched data set, only a subsample from the Japanese study was used due to the matching procedure. After excluding the oldest-old participants, the matched Japanese sample was younger, and had lower educational status and IADL compared with their unmatched counterparts [*t*(2,161) = 4.67, *p* < .001; *t*(2,152) = 3.06, *p* = .002; and *t*(217.68) = 4.40, *p* < .001, respectively].

### Measures

#### Predictors

Sociodemographic, social, and health resources were assessed as potential VOL predictors. Age (in years), gender (0 = *male*, 1 = *female*), and education (in years of schooling) were included as sociodemographic predictors. Four social predictors were assessed: we asked participants whether they lived with a spouse and if they lived alone (0 = *no*, 1 = *yes* for both variables); number of children was also assessed. In addition, we asked how often participants were in contact with others living in other households such as non-coresident children, friends, and neighbors (0 = *less than once per week*, 1 = *once per week*, 2 = *more than twice per week*). Three health-related predictors were measured: subjective hearing, subjective health, and IADL. We asked participants to evaluate their hearing using a 3-point scale ranging from 0 (*poor*) to 2 (*good*) and to evaluate their general health on a 4-point scale ranging from 0 (*poor*) to 3 (*excellent*). We also assessed four IADLs (shopping, cooking, organizing finances, and using public transportation); individuals indicated whether they were able to perform each activity (0 = *no*, 1 = *yes*).

#### Valuation of life

VOL was measured using five items from the Positive Valuation of Life scale (Lawton et al., 1999, 2001). The original scale consists of 13 items and includes five positive aspects of life identified in the previous psychological literature: hope, purpose, self-efficacy, persistence, and futurity. Each item reflects a global evaluation toward one’s life. Because only one item per VOL aspect were shared between the German and Japanese studies, the total of five items were analyzed in this study: whereas all items from the VOL scale were included in the German study, only a limited number of items were measured in the Japanese study. The items were “I feel hopeful right now” for hope; “I meet the goals that I set for myself” for purpose; “I can think of many ways to get out of a jam” for self-efficacy; “Even when others get discouraged, I know I can find a way to solve the problem” for persistence; and “Each new day I have much to look forward to” for futurity. Participants were asked to indicate how well the items described themselves using a 3-point Likert scale (Do these items describe yourself?; 0 = *not at all*, 0.5 = *somewhat*, 1 = *completely*). The Positive VOL scale has been validated in both countries (Jopp et al., 2008; Nakagawa et al., 2013).

Testing the measurement equivalence of VOL across the countries, although fit indices of the configural invariance model were significantly better than those of the metric invariance model [χ^2^(10) = 23.50, *p* < .01, comparative fit index (CFI) = .97, root mean square error of approximation (RMSEA) = .05, Akaike information criterion (AIC) = 83.50 for the configural invariance model, and χ^2^(14) = 32.59, *p* < .01, CFI = .96, RMSEA = .05, AIC = 84.59 for the metric invariance model], the overall fit indices of both models were equally acceptable and thus results indicated measurement equivalence across the two countries. Cronbach’s alpha coefficients were good (Germany: .74 for total sample, .64 for young-old, and .79 for old-old; Japan: .73 for total sample, .74 for young-old, and .70 for old-old).

### Statistical Analysis

The statistical analysis consisted of four steps. First, we performed chi-square tests separately for the young-old and old-old to compare the distributions of the categorical variables between the two countries and conducted 2 (country: Germany vs Japan) × 2 (age group: young-old vs old-old) ANOVAs to examine country and age differences in mean values for the continuous variables. Second, we examined correlations between variables for each country. Third, multiple hierarchical regression analyses including all predictors concurrently were performed for each country and age group. Fourth, based on Hayes (2013), we performed multiple regression analyses that tested three-way and two-way interactions between predictors, country, and age group, to confirm potential differences in the explanatory values of the predictors according to country and age group.

## Results

### Descriptive Statistics

Both German and Japanese samples were quite similar, and differed only with respect to a few variables, including social and health variables (Table 1): a chi-square test indicated that a higher number of old-old Germans lived alone compared with their Japanese counterparts, and a two-way ANOVA revealed that Germans had more frequent social contact compared with Japanese participants. Regarding health factors, Japanese participants reported better subjective hearing and subjective health compared with German participants.

**Table 1.** Sample Characteristics of Key Variables in the German and Japanese Participants

			Germany						Japan				
	Total (*N* = 257)		Young-old (*n* = 131)		Old-old (*n* = 126)		Total (*N* = 248)		Young-old (*n* = 132)		Old-old (*n* = 116)		Sample differences tests^a^
Variable	*M*	*SD*	*M*	*SD*	*M*	*SD*	*M*	*SD*	*M*	*SD*	*M*	*SD*	
Age (years)	74.21	5.67	69.36	2.85	79.25	2.66	74.81	5.09	70.20	1.30	80.05	1.30	Country: *F*(1, 501) = 18.22, *p* < .001, part *η*^2^ = .04; Age: *F*(1, 501) = 2601.84, *p* < .001, part *η*^2^ = .84
Gender (% female)	46.3		46.6		46.0		48.0		41.7		55.2		Young-old: χ^2^(1) = 0.64, n.s., *φ* = .05; Old-old: χ^2^(1) = 2.01, n.s., *φ* = .09
Education (years)	11.77	3.10	11.96	3.11	11.57	3.10	12.01	2.33	12.15	2.07	11.84	2.58	Country: *F*(1, 501) = 0.89, n.s., part *η*^2^ = .00; Age: *F*(1, 501) = 2.02, n.s., part *η*^2^ = .00
Living with spouse (% yes)	65.8		73.3		57.9		69.8		78.8		59.5		Young-old: χ^2^(1) = 1.09, n.s., *φ* = .06; Old-old: χ^2^(1) = 0.06, n.s., *φ* = .02
Living alone (% yes)	26.5		22.1		31.0		16.9		14.4		19.8		Young-old: χ^2^(1) = 2.64, n.s., *φ* = .10; Old-old: χ^2^(1) = 3.92, *p* < .05, *φ* = .13
Number of children	1.81	1.08	1.83	1.19	1.79	1.44	1.79	0.94	1.72	0.98	1.86	0.89	Country: *F*(1, 501) = 0.03, n.s., part *η*^2^ = .00; Age: *F*(1, 501) = 0.22, n.s., part *η*^2^ = .00
Social contacts (0–2)^b^	1.73	0.48	1.77	0.44	1.68	0.52	0.95	0.90	0.99	0.90	0.91	0.89	Country: *F*(1, 501) = 76.21, *p* < .001, part *η*^2^ = .23; Age: *F*(1, 501) = 1.89, n.s., part *η*^2^ = .00
Subjective hearing (0–2)^b^	1.04	0.45	1.10	0.44	0.97	0.45	1.33	0.76	1.39	0.75	1.26	0.80	Country: *F*(1, 501) = 26.42, *p* < .001, part *η*^2^ = .05; Age: *F*(1, 501) = 5.30, *p* < .05, part *η*^2^ = .01
Subjective health (0–3)^b^	1.52	0.67	1.63	0.60	1.41	0.72	1.95	0.64	1.97	0.48	1.92	0.70	Country: *F*(1, 501) = 54.34, *p* < .001, part *η*^2^ = .10; Age: *F*(1, 501) = 5.06, *p* < .05, part *η*^2^ = .01
IADL (0–4)^b^	3.50	0.97	3.82	0.52	3.17	1.20	3.57	0.82	3.77	0.60	3.34	0.96	Country: *F*(1, 501) = 0.68, n.s., part *η*^2^ = .00; Age: *F*(1, 501) = 48.92, *p* < .001, part *η*^2^ = .09
VOL (0–5)^b,c^	4.10	1.13	4.23	0.96	3.95	1.27	3.34	1.18	3.24	1.16	3.45	1.20	Country: *F*(1, 501) = 53.22, *p* < .001, part *η*^2^ = .10; Country × Age: *F*(1, 501) = 5.69, *p* < .05, part *η*^2^ = .01

*Note:* IADL = instrumental activities of daily living; part *η*^2^ = partial *η*^2^; n.s. = not significant, *SD*, standard deviation; VOL = valuation of life.

^a^Two-way ANOVAs examined country, age group, and country × age group interaction effects. Significant and nonsignificant main effects are reported, but only significant interaction effects are shown. Chi-square tests tested the effect of country on frequencies separately for young-old and old-old participants.

^b^Higher values indicate more frequent social contacts, better health, and higher levels of VOL.

^c^Old-old participants showed lower levels of VOL compared with young-old participants in Germany, but age difference was not found in Japan.

Regarding mean scores of VOL, we found a significant main effect of country and a two-way interaction between country and age group. Overall, German participants showed higher levels of VOL relative to Japanese participants. Follow-up analysis for the significant interaction effect showed that there was no age difference among the Japanese participants, whereas there was a tendency for old-old Germans to have lower VOL levels compared with the young-old Germans.

### Zero-Order Correlations According to Country

Comparing potential VOL predictors between the two countries, zero-order correlation patterns showed both similarities and differences (Supplementary Table 1). In terms of similarities, VOL was correlated in both German and Japanese participants with social contacts (*r* = .27, *p* < .001 and *r* = .19, *p* < .01, respectively), subjective health (*r* = .43 and *r* = .26, *p*s < .001, respectively) and IADL (*r* = .47, *p* < .001 and *r* = .19, *p* < .01, respectively). In terms of differences, VOL was significantly correlated with age (*r* = −.17, *p* < .01) and education (*r* = .13, *p* < .05) in the German sample, and with number of children (*r* = .13, *p* < .05) in the Japanese sample.

Since correlations indicated a substantial overlap between living with a spouse and living alone in both countries (Germany: *r* = −.69 and Japan: *r* = −.83, *p*s < .001), we included only the variable “living alone” in the subsequent regression analyses, to prevent multicollinearity.

### Predictors of VOL

Multiple hierarchical regression analyses were conducted with the sociodemographic, social, and health predictors, beginning with the country-specific total samples and then splitting them according to age group (Table 2).

**Table 2.** Multiple Hierarchical Regression Analyses Predicting VOL According to Country and Age Group

			Total				Young-old				Old-old			
Country	Predictor block	Variable	*B*	*SE*	*β*	∆*R*^2^	*B*	*SE*	*β*	∆*R*^2^	*B*	*SE*	*β*	∆*R*^2^
Germany	1: Sociodemographic	Age	0.01	0.01	.04	.00	−0.01	0.03	−.04	.01	0.01	0.04	.02	.02
		Gender^a^	−0.11	0.13	−.05		−0.12	0.16	−.06		−0.06	0.20	−.02	
		Education	0.01	0.02	.03		−0.02	0.02	−.05		0.05	0.03	.12	
	2: Social	Living alone	−0.09	0.15	−.04	.04**	0.26	0.20	−.11	.05*	0.04	0.22	.01	.09**
		Number of children	0.12	0.06	.11*		−0.07	0.08	−.08		0.28	0.08	.26***	
		Social contactb	0.38	0.13	.16**		0.43	0.17	.20*		0.31	0.19	.13	
	3: Health	Subjective hearing^b^	−0.10	0.13	−.04	.22***	−0.32	0.17	−.15	.16***	0.25	0.22	.09	.27***
		Subjective health^b^	0.42	0.10	.25***		0.46	0.13	.29***		0.26	0.16	.15	
		IADL^b^	0.43	0.07	.37***		0.42	0.15	.23**		0.46	0.10	.44***	
	*R* ^2^ total					.33***				.30***				.41***
Japan	1: Sociodemographic	Age	0.02	0.02	.10	.03*	−0.03	0.08	−.03	.04	−0.11	0.09	−.12	.02
		Gender^a^	0.19	0.16	.08		0.26	0.21	.11		−0.07	0.24	−.03	
		Education	0.07	0.03	.13*		0.11	0.05	.19*		0.04	0.04	.08	
	2: Social	Living alone	0.09	0.20	.03	.03*	0.28	0.28	.09	.07*	0.30	0.27	.10	.04
		Number of children	0.15	0.08	.12*		0.04	0.10	.04		0.29	0.13	.21*	
		Social contactb	0.15	0.08	.12		0.32	0.11	.25**		−0.07	0.13	−.05	
	3: Health	Subjective hearing^b^	0.04	0.09	.03	.08***	0.15	0.13	.10	.13***	−0.10	0.14	−.07	.08*
		Subjective health^b^	0.45	0.12	.24***		0.54	0.16	.27**		0.44	0.18	.26*	
		IADL^b^	0.17	0.10	.12		0.39	0.16	.20*		0.09	0.13	.07	
	*R* ^2^ total					.15***				.26***				.17*

*Note:* Germany: total: *N* = 257; young-old: *n* = 131; old-old: *n* = 126. Japanese: total: *N* = 248; young-old: *n* = 132; old-old: *n* = 116. *∆R*^2^*=* independent explained variances of each predictor block determined by entering all the predictors in the last step of a multiple hierarchical analysis. IADL = instrumental activities of daily living; *SE* = standard error; VOL = valuation of life.

^a^0 = *male*, 1 = *female*.

^b^Higher values indicate more frequent social contacts and better health.

*p < .05. **p < .01. ***p < .001.

Considering the country-specific total samples, the proportion of the variance in VOL explained in the German sample was twice as large as the variance explained in the Japanese sample (*R*^2^ = .33 and *R*^2^ = .15, respectively). In both countries, parallel predictive patterns occurred, with number of children and subjective health being significant predictors with similar beta values (Germany: β = .11, *p* < .05 and β = .25, *p* < .001, respectively; Japan: β = .12, *p* < .05, and β = .24, *p* < .001, respectively). Differences in predictive patterns were also observed. In particular, education was a significant predictor in the Japanese sample (β = .13, *p* < .05) but not in the German sample. In contrast, social contact and IADL were significant predictors in the German sample (β = .16, *p* < .01, and β = .37, *p* < .001, respectively) but not in the Japanese sample. Furthermore, despite being significant predictors in both samples, health variables explained a much higher proportion of unique variance in the German (22%) than in the Japanese sample (8%).

Next, we concentrated on the younger participants. In both German and Japanese young-olds, the proportions of overall variance explained by the models were comparable (Germany: *R*^2^ = .30, Japan: *R*^2^ = .26). Social contact, subjective health, and IADL were significant VOL predictors in both countries (Germany: β = .20, *p* < .05, β = .29, *p* < .001, and β = .23, *p* < .01, respectively; Japan: β = .25, *p* < .01, β = .27, *p* < .01, and β = .20, *p* < .05, respectively). In both countries, the unique variance explained by social and health predictors were similar (Germany: *R*^2^ = .05, and *R*^2^ = .16, respectively; Japan: *R*^2^ = .07, and *R*^2^ = .13, respectively). One difference occurred in that education was a significant predictor in the Japanese (β = .19, *p* < .05), but not the German young-old group.

When addressing the old-old group, regression models were significant in both countries, but the variance explained in VOL was much larger in the German (*R*^2^ = .41), relative to the Japanese (*R*^2^ = .17) old-old group. Substantial differences were also observed for the unique variance explained by the health variables, being more than three times larger in the Germans (27%) than in the Japanese (8%). In both countries, number of children was a significant VOL predictor, and showed comparable beta values (Germany: β = .26, *p* < .001; Japan: β = .21, *p* < .05). The predictive values of subjective health (Germany: β = .15, not significant (n.s.); Japan: β = .26, *p* < .05) and IADL (Germany: β = .44, *p* < .001; Japan: β = .07, n.s.) differed between the two countries, but follow-up analyses did not confirm any significant differences in the regression coefficients (see below).

To confirm whether the regression coefficients differed significantly among the four groups (i.e., 2 countries × 2 age groups), we conducted follow-up regression analyses, including the following three-way and two-way interaction terms simultaneously: predictor × country × age group, predictor × country, predictor × age group, and country × age group. For sociodemographic predictors, the interaction between education and country was significant (*p* < .05), validating our prior finding that education was positively associated with VOL only in the young-old Japanese (Table 2). Regarding social predictors, the interaction between number of children and age group was significant (*p* < .01), supporting the finding that number of children was positively related in the old-old, but not in the young-old. Lastly, in terms of health predictors, no significant interaction was detected, and only the main effects of subjective health and IADL were significant (*p* < .01 and *p* < .05, respectively), indicating that subjective health and IADL had similar effects on VOL regardless of culture and age. After including all the predictors, the interaction between country and age was not significant, but culture was still significantly associated with VOL (*p* < .001), which indicated that, whereas the predictors explained age differences in levels of VOL found in Germany, they did not fully explain country differences in levels of VOL.

## Discussion and Implications

To better understand how individuals perceive or make sense of their life as valuable, the present study examined cultural and age-related variations in mean levels and predictors of active attachment to life, assessed via the VOL construct (Lawton et al., 1999, 2001), in the young-old and old-old individuals from Germany and Japan representing independence- and interdependence-oriented cultures, respectively. This study supported the assumption and revealed that, as a prior study indicated (Fung, Stoeber, Yeung, & Lang, 2008), the Japanese participants had smaller nonfamily networks compared with the German participants, and co-residence with others was more common in the old-old Japanese relative to their German counterparts. This preference for ingroup members is considered to be associated with interdependent values (Brewer & Chen, 2007).

To our knowledge, this is the first investigation to examine the role of culture and its intersections with aging in influencing the VOL construct. Our results showed both cultural and age-related differences in mean levels and predictors of VOL, which were overall in line with cross-cultural and life-span theories reviewed in the introduction (Carstensen, 2006; Markus & Kitayama, 1991). This study suggested that both culture and aging serve a unique role, but that their interactions were absent: there are cultural variations, both in terms of levels and predictors of VOL, but age may play a similar role for the predictive differences across cultures. Let us summarize our main findings.

### Mean levels of VOL, Culture, and Age

According to the cross-cultural theory of self-construals (Markus & Kitayama, 1991), we first hypothesized that mean levels of VOL would be higher in Germany than in Japan, since individuals with an independent self-construal would tend to appreciate personal capacity of maintaining autonomy in later life and thus health resources would be more important in independent cultures. Our results showed that German older adults exhibited higher levels of VOL relative to their Japanese counterparts (Table 1), but this cultural difference persisted even after controlling for its potential predictors including health factors, which indicates that being attached to his/her life itself may be more valued in independent cultures. An earlier study also presented that multiple well-being indicators (e.g., life satisfaction and psychological well-being) were still higher in the United States than in Japan after controlling for predictors such as personality (Kitayama, Karasawa, Curhan, Ryff, & Markus, 2010). Given these results, VOL encompassing well-being constructs such as purpose and self-efficacy could be deeply rooted in independent values, and thus individuals from independent cultures might strongly value the positive appraisals of their life for themselves.

We further found age differences in attachment to life but only for Germany: there were slightly lower levels of VOL among old-old Germans compared with their young-old counterparts, whereas there were no age differences in Japan. This seems not to be due to measurement issues, given the measurement equivalence across countries and similar standard deviations across all four comparison groups. In fact, our follow-up analyses indicated that the age differences observed in the German sample could be explained by potential predictors. Although we did not find evidence of greater age differences in health resources in Germany relative to in Japan, loss of autonomy might be more salient in very late life in an independence-oriented culture, which could endanger Germans’ attachment to life.

### Predictors of VOL, Culture, and Age

According to the socioemotional selectivity theory (Carstensen, 2006) in addition to the cross-cultural theory, we hypothesized both cultural and age-related variations in predictive patterns of perceiving life as valuable. Our follow-up analyses provided partial support for the moderating role of culture and age: sociodemographic resources were more valued in an interdependent culture, and social resources were of particular importance in very late life. Besides, intersections between culture and aging were not observed.

As we secondly hypothesized, the present study showed that sociodemographic resources were more important in Japan relative to in Germany: education exerted a significant effect on VOL in the young-old Japanese, but not German participants. Previous studies also indicate that Easterners emphasize social standing for evaluating one’s worth to a greater extent relative to Westerners (Twenge & Campbell, 2002), which in turn could have caused the stronger link between education and VOL. Thus, one’s position in social hierarchies may be more salient to self-evaluation in interdependent cultures relative to in independent ones.

Furthermore, in line with our third hypothesis, we found an age-related shift in predictive values in social resources in both countries: number of children was as a significant VOL predictor in the old-old, but not in the young-old. This result was consistent with the life-span theory stating that older adults prefer intimate social contact with close others such as children, to peripheral social relationships with other individuals such as neighbors (Carstensen, 2006). These age-differential motivations also appeared to be reflected in our results, and very old individuals’ attachment to their life could be more closely related to having close social partners.

However, social and health resources equally mattered in both countries. These results contradicted our hypotheses that social resources would be more important in interdependent cultures and that health resources would matter more in independent cultures. Thus, cultural variations in predictors of VOL were rather modest. Regarding social resources, with the exception of the old-old Japanese sample, the social predictors explained a significant proportion of the individual differences in VOL in the regression analyses. The lack of predictive value in the old-old Japanese may be due to the fact that we examined the quantity, but not quality, of social relationships. In a prior study, relationship quality, as measured by positive and negative responses from ingroup members, was a stronger predictor of well-being in the Japanese relative to in Americans (Kitayama et al., 2010). An alternative possibility is that social factors may play a special role in valuing life, and that feeling attached to one’s life can be more strongly determined by a universal need for relatedness (Deci & Ryan, 2000). This could explain why social factors differed less across cultures.

The importance of health resource was evident irrespective of culture and age: although health was not a significant predictor in the old-old Japanese, no significant interaction confirmed any differences in predictive validity of health. Earlier studies also reported that health was significantly associated with VOL (Gitlin et al., 2016; Jopp et al., 2008; Lawton et al., 2001; Nakagawa et al., 2013). These results lend support to life-span theory assumptions (e.g., Baltes & Smith, 2003), suggesting that health declines are key issues to adapt to later life. Findings from centenarian studies support the fact that health remains the number one challenge reported (Jopp et al., 2016), although its links to well-being constructs may vary (Jopp & Rott, 2006). Following our results, age-related losses in health seem to impose a limitation on the perceived value of life in very late life across cultures (Baltes & Smith, 2003; Jopp et al., 2008).

### Limitations

Several limitations of the current study warrant consideration. First, the response rates were low particularly in Japan. Furthermore, given that German participants completed a mail survey, and Japanese participants attended community centers for assessment in person, Japanese participants might be more positively selected as they had to be more motivated to attend the venue to participate. These features limit the generalizability of the present results.

Second, we could select only a subset of available variables. For example, we included hearing ability in the analysis but could not consider vision as a predictor, because assessment differed between the two countries. Furthermore, while we highlighted the potential role of culture, we did not directly measure cultural values, namely independence versus interdependence orientations. Use of parallel research designs and protocols in future studies would help to increase our understanding of the role of culture in VOL.

Third, even though we were able to show that our VOL scale had acceptable measurement equivalence across cultures, we used only a limited number of items from the Positive VOL scale (i.e., 5 out of 13) on the basis of the Japanese study. In addition, potential predictors explained only a much smaller amount of variance in VOL in the old-old Japanese (17%) compared with their German counterparts (41%). Since VOL was developed based on independent values such as personal autonomy, the construct could miss social aspects which are more important among the older Japanese cohorts who might more deeply internalize interdependent values. Future studies should further explore cultural meanings of well-being to understand the variation and commonality of VOL across cultures.

### Implications

Old and very old individuals from Germany, an independence-oriented culture, were found to perceive their life as more valuable relative to their counterparts from Japan, an interdependence-oriented culture, which may be related to the nature of the VOL construct deeply rooted in independent values such as personal autonomy. Findings further indicate both cultural and age-related variations in predictive patterns for VOL: whereas the importance of social status can vary depending on cultures, aging processes may universally influence how people value their life. However, the cultural differences were rather modest, and intersections between culture and aging were not found. Despite these results, this study suggests that cultural values deserve much more attention in studying aging processes and that professional care providers and family members will benefit from these findings to help older adults to feel that his/her life is meaningful and worth living. Clearly, more research and theory development are needed to better understand how individuals make sense of their life with advancing age across various cultures.

## Supplementary Material

Supplementary data are available at *Innovation in Aging* online.

Supplementary Table 1

## Funding

This work was supported by the program for promoting the enhancement of research universities from Osaka University. The German study was funded by the city council of Darmstadt, Germany (co-principal investigators: Frank Oswald, Christoph Rott, and Hans-Werner Wahl), and the Japanese study was supported by Japan Society for the Promotion of Science KAKENSHI (JP21330152).

## Conflict of Interest

None reported.
