# Supplementary material for: Valuation of Life Among Old and Very Old Adults: Comparison Between Germany and Japan
Source: Innov Aging. 2018 Jul 11;2(2):igy020. doi: 10.1093/geroni/igy020 (PMC6177057; doi:10.1093/geroni/igy020)
Supplement: Supplementary Table 1 [file igy020_suppl_supplementary-table-1.docx]

**Supplementary Table 1**. Intercorrelations Between VOL Predictors According to Country (Germany Above and Japan Below the Diagonal Line)

|  | 1 | 2 | 3 | 4 | 5 | 6 | 7 | 8 | 9 | 10 | 11 |
| --- | --- | --- | --- | --- | --- | --- | --- | --- | --- | --- | --- |
| 1. Age | − | .01 | −.10 | −.19** | .13* | −.02 | −.11 | −.10 | −.18** | −.38*** | −.17** |
| 2. Gender^a^ | .14* | − | −.08 | −.35*** | .33*** | .07 | .06 | .08 | −.05 | .00 | −.06 |
| 3. Education | −.09 | −.15* | − | .07 | .04 | −.04 | .10 | −.02 | .15* | .15* | .13* |
| 4. Living with spouse | −.23*** | −.35*** | −.00 | − | −.83*** | .09 | −.03 | .00 | .11 | .00 | .07 |
| 5. Living alone | .11 | .23*** | .03 | −.69*** | − | −.12 | .08 | .01 | −.03 | .07 | −.03 |
| 6. Number of children | .03 | −.10 | −.03 | .14* | −.17** | − | .04 | −.05 | −.05 | −.11 | .07 |
| 7. Social contact^b^ | −.05 | .20** | −.05 | −.09 | .08 | .15* | − | .08 | .24*** | .15* | .27*** |
| 8. Subjective hearing^b^ | −.09 | .03 | −.10 | −.05 | −.02 | .07 | −.04 | − | .17** | .18** | .07 |
| 9. Subjective health^b^ | −.04 | −.16* | .01 | .08 | −.08 | .03 | .10 | −.01 | − | .43*** | .43*** |
| 10. IADL^b^ | −.25*** | .14* | .00 | −.10 | .18** | −.00 | .20** | −.01 | .23*** | − | .47*** |
| 11. VOL^b^ | .06 | .07 | .10 | −.04 | .05 | .13* | .19** | .01 | .26*** | .19** | − |

*Note*. Germany: *N* = 257, Japan: *N* = 248; ****p* < .001, ***p* < .01, **p* < .05.

^a^ 0 = male, 1 = female. ^b^ Higher values indicate more frequent social contacts, better health, and higher levels of VOL.

IADL = Instrumental Activities of Daily Living; VOL: Valuation Of Life.
